# Structural Characterization of the Self-Association of the Death Domain of p75^NTR^

**DOI:** 10.1371/journal.pone.0057839

**Published:** 2013-03-05

**Authors:** Qianhui Qu, Jun Chen, Yizhi Wang, Wenjun Gui, Li Wang, Zusen Fan, Tao Jiang

**Affiliations:** 1 National Key Laboratory of Biomacromolecules, Institute of Biophysics, Chinese Academy of Sciences, Beijing, China; 2 Key Laboratory of Infection and Immunity, Institute of Biophysics, Chinese Academy of Sciences, Beijing, China; 3 Graduate University of Chinese Academy of Sciences, Beijing, China; University of Washington, United States of America

## Abstract

The neurotrophin receptor p75^NTR^ conveys multiple signals via its intracellular death domain. However, how the death domain is activated and interacts with downstream adaptors remains unclear. Here, we report two crystal structures of the p75^NTR^ death domain in the form of a non-covalent asymmetric dimer and a Cys379-Cys379 disulfide bond linked symmetric dimer, respectively. These two dimer arrangements have not previously been observed in other death domain-containing proteins. Further analysis shows that both the Cys379-Cys379 disulfide linked and non-covalent full-length p75^NTR^ dimers are present on the cell surface. These observations suggest that various oligomers may exist simultaneously on the cell surface, and that p75^NTR^ activation and signalling may be modulated by neurotrophins or other factors via inducing a shift of the equilibrium between different oligomeric states.

## Introduction

Neurotrophins are a family of secreted growth factors essential for early stage innervation and maintenance of the adult vertebrate nervous system. They interact with two different types of cell surface receptors, the selective Trk receptor tyrosine kinases and p75^NTR^, which binds to all neurotrophins with equal affinity [1], [2]. Unlike the Trk receptors, which possess characteristic tyrosine kinase domains, p75^NTR^ lacks intrinsic kinase activity. Glycosylated extracellular cysteine-rich domains serve as the ligand-binding site of p75^NTR^, while its intracellular region consists of a flexible juxtamembrane segment (residues 280–333), a globular death domain (residues 334–418), and a short tail (residues 419–425) [3], [4]. In addition to its expression in the central and peripheral nervous systems, p75^NTR^ is also widely expressed in non-neuronal cells, including immune cells, smooth muscle and epithelial cells, and fibroblasts [5]. In addition to neurotrophins, p75^NTR^ can recognise a number of novel ligands such as β-amyloid peptides, prion peptides and myelin-associated inhibitors, in cooperation with their related co-receptors [6], [7], [8], [9], [10]. Signals in distinct pathways mediated by p75^NTR^ are conveyed by the recruitment and release of multiple cytoplasmic adaptor proteins such as neurotrophin receptor interacting factor (NRIF), neurotrophin receptor interacting MAGE homolog (NRAGE) and tumor necrosis factor receptor (TNFR) associated factor 6 (TRAF6), and result in the activation of a wide array of downstream signalling molecules (e.g., nuclear factor kappa B (NF-kB), c-jun N-terminal kinase (JNK) or caspases) [11], [12]. Through these downstream events, p75^NTR^ regulates various cellular functions including cell apoptosis, survival, neurite outgrowth and synaptic plasticity, making it an important target for the treatment of neurodegenerative and other diseases [13], [14], [15], [16], [17], [18].

The monomeric form of the intracellular death domain of p75^NTR^, solved by NMR, displays a folding module that is similar to death domain superfamily members such as Fas and TNFR, in spite of low sequence identity [3]. However, these proteins show different oligomerization patterns. Fas and TNFR bind trimeric ligands to induce the trimer- or oligomerization of their intracellular death domains, while biochemical data and the structures of the p75^NTR^ ectodomain complex with NT3 [19] and proNGF [20] have demonstrated that p75^NTR^ binds dimeric neurotrophins and that dimerization of the p75^NTR^ intracellular region is functionally significant [21]. Therefore, the association mechanism of the death domain of p75^NTR^ is likely to be different from that of Fas and TNFR. Recently, Vilar and colleagues reported that p75^NTR^ could exist as a disulfide bond-linked dimer before ligand binding via a highly conserved Cys257 residue in its transmembrane region, and the dimer proportion in transfected cells didn't change upon NGF binding [22]. Fluorescence resonance energy transfer (FRET) experiments also revealed that close association of p75^NTR^ intracellular domains was transiently disrupted by conformational changes that occured upon ligand binding. These results suggest that neurotrophins activate p75^NTR^ by a mechanism involving the rearrangement of intracellular domains, in which Cys257 acts as a fulcrum, mediating the propagation of conformational changes (“Snail-Tong”model [22]). However, how the p75^NTR^ death domain associates with itself or with other proteins is still unclear.

**Figure 1:**
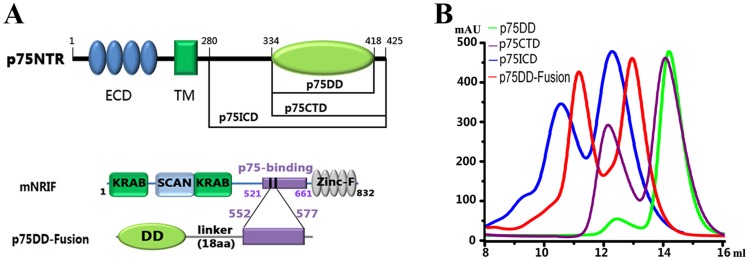
Solution features of different constructs of p75^NTR^ intracellular region. (A) Schematic diagram of the p75^NTR^ constructs used in this work. (B) Size-exclusion chromatography profiles of these constructs on Superdex 75 column. All proteins elute as mixture of dimer and monomer, while the dimer portion of p75DD is not that obvious.

To gain insight into the mechanism of p75^NTR^ regulation, we determined the structural and functional properties of the following truncations involving the rat p75^NTR^ intracellular region: p75DD (residues 334–418), p75CTD (334–425), p75ICD (280–425), and p75DD-Fusion (a chimera of the p75^NTR^ death domain and a p75-interacting peptide from NRIF) (Fig. 1A). By introducing cysteine modifications using 5,5′-dithio-bis(2-nitrobenzoic acid) (DTNB), we obtained the crystal structures of the p75DD and p75DD-Fusion proteins. In the crystal structure of p75DD, we observed an asymmetric dimer in which the H5 and H6 helices of one death domain monomer juxtaposed against the H3 helix of another. The structure of the p75DD-Fusion protein revealed that the p75^NTR^ death domain presented as a novel symmetric dimer linked via an inter-chain disulfide bond between Cys379 residues. Further *in vitro* binding experiments showed that one downstream adaptor protein, RIP2, could only interact with the monomer, not the symmetric dimer of the p75^NTR^ death domain; while a synthetic NRIF peptide bound to both monomer and dimer without significant difference in apparent affinity. Biochemical analyses indicated the Cys379-Cys379 disulfide linked p75^NTR^ dimer could exist on the 293T cell surface. Considering these data together with previous biochemical evidence, we proposed a model that multiple oligomerization patterns of p75^NTR^ present on the cell surface may confer different regulation levels to the p75^NTR^ signalling pathway.

## Results

The oligomeric behavior of the four p75^NTR^ constructs in solution is shown in Figure 1B; all constructs are present as a mixture of monomers and dimers. Intriguingly, p75DD exists less frequently as a dimer, implying that the short C-terminal tail (residues 419–425) in p75^NTR^ is important for dimer formation. We failed to obtain crystals until the proteins were treated with DTNB, a cysteine-modifying reagent that generates a homogeneous oxidised state and stabilises local fluctuations [23]. After treatment, suitable p75DD and p75DD-Fusion protein crystals were obtained for structural analysis.

### A unique asymmetric interaction occurs between death domains of p75^NTR^

**Figure 2:**
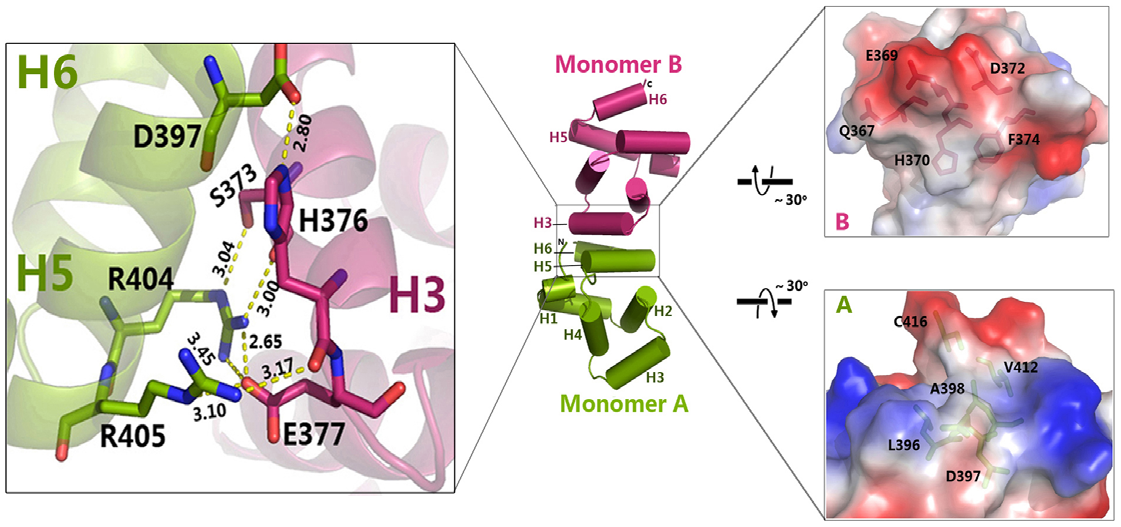
Asymmetric dimeric interface in the p75DD crystal lattice. Details of the asymmetric dimer interface are shown in the left expanded view. The interfacial electrostatic distribution is shown in the two right expanded views, with residues involved in hydrophobic interactions highlighted as sticks.

The structure of p75DD was solved by molecular replacement using the NMR structure (PDB 1NGR) as the search model. After model building and refinement, we were able to trace amino acids 334–417 of the death domain. The overall fold of the p75DD monomer is similar to the NMR structure with a small RMSD value (1.44 Å) of Cα traces (Fig. S1A). Briefly, the N-terminal segment is close to the C-terminus, and six helices connected by short loops encircle into a tight globular structure, with the third helix (H3) extending slightly away. In the crystal lattice, neighbouring death domains stack to form asymmetric dimers, with the H5 and H6 helices of one monomer (A) juxtaposed against the H3 helix of the other monomer (B), forming interfaces enriched with polar and hydrophobic interactions, e.g. R404 from monomer A interacts with S373, H376 and E377 of monomer B (Fig. 2), in agreement with speculation that the H5 helix is important for the association of p75^NTR^ with other proteins [24]. DTNB-modified Cys379 and Cys416 in p75DD stabilise the monomer structure, while these residues are not involved in monomer-monomer interactions (Figs. S1B and S1C).

The death domain, a versatile protein interaction module, plays an essential role in the assembly and activation of apoptotic and inflammatory complexes such as the DISC, PIDDosome and TLR/IL-1 receptor complex [25]. While the monomeric structures of most of these death domains have been solved via NMR under acidic conditions that might disrupt the oligomeric capacity of the proteins, several death domain complex structures have been elucidated by X-ray crystallography [26], [27], [28], [29], [30]. An intriguing feature prevalent among these complex structures is that death domains, such as those in the Pelle-Tube complex [26] and Fas/FADD oligomer [30], contact each other in an asymmetric fashion. Different monomer-monomer interfaces between death domains have been categorised into the following three types based on their contact region: type I (H1/H4: H2/H3), type II (H4/H4–H5 loop: H6/H5–H6 loop) and type III (H3: H1–H2 loop/H3–H4 loop) [27], [31]. In our p75DD structure, the size of buried solvent accessible area (320 Å^2^ [32]; Shape complementarity value = 0.75 [33]) of the asymmetric dimer was comparable to that of other death domain complexes, but it had a novel H5/H6:H3 asymmetric interface (Fig. S2).

### The symmetric dimer of the p75^NTR^ death domain is linked via an inter-chain disulfide bond

The p75DD-Fusion protein was initially designed as a chimera between the p75^NTR^ death domain and a p75^NTR^-interacting peptide from NRIF, connected by an 18-amino-acid linker (Fig. 1A). NRIF is the first protein identified to associate with the intracellular domain of p75^NTR^ and mediates p75^NTR^-dependent neuronal death [34], [35]. A previous study demonstrated that the p75^NTR^-binding region spans residues 521–661, a flexible loop structure, in NRIF [34]. We constructed this loop region of NRIF preceded by a glutathione S-transferase tag and co-expressed it with the His_6_-tagged p75DD protein in *E. coli*. However, progress was hampered by severe proteolysis of NRIF. After many attempts, we found that a short NRIF peptide spanning residues 552–577 (determined by mass spectrometry) co-eluted with the p75DD protein during size exclusion chromatography. We then generated the p75DD-Fusion protein and successfully crystallised the dimer fraction (Figs. S3A and S3B). However, mass spectrometry indicated that, unfortunately, only the p75^NTR^ death domain and a portion of the linker residues (GSGSGSGS) were retained in p75DD-Fusion crystals (Fig. S3C). Even so, only the death domain portion (residues 335–417) was visible in the final electron density map. For ease of description, the structure solved was still named p75DD-Fusion.

**Figure 3:**
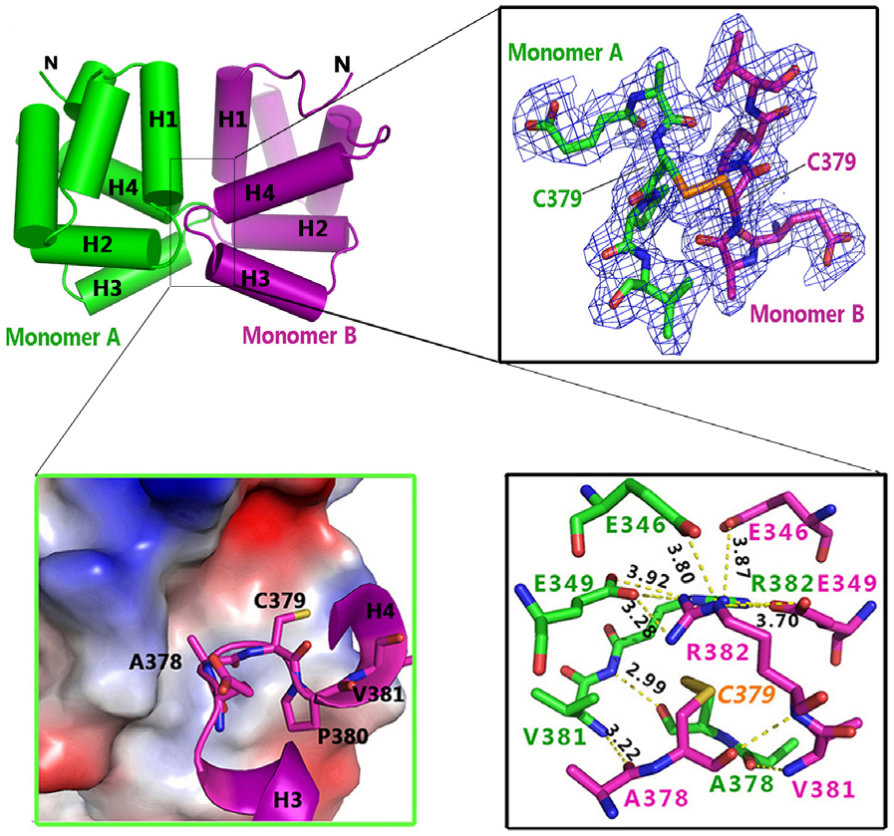
Disulfide linked symmetric dimer of p75^NTR^ death domain. The 2Fo-Fc electron density (contour σ = 1.6) shows the inter-chain disulfide bond (orange) formed by the Cys379 residues from both monomers (right expanded view). Details of the symmetric dimer interface are shown below in two expanded views. The inter-chain disulfide bond formed between Cys379 residues is highlighted as orange sticks.

Asymmetric units of the p75DD-Fusion crystal contain two monomers symmetrically related by a non-crystallographic two-fold rotation axis. Inspection of the buried interface (552 Å^2^) shows that an inter-chain disulfide bond is formed between the Cys379 residues from each monomer (Fig. 3), while Cys416 is modified by DTNB and is not involved in monomer-monomer interactions (Fig. S1B). Several residues distributed on the interface interact via hydrogen bonds or hydrophobic interactions through both main chain and side chain residues. For example, Arg382 on the amino side of H4 interacts with Glu346 and Glu349 from the first helix. The loop region between H3 and H4 of one death domain (monomer B) protrudes into the H1–H2 and H3–H4 regions of the other (monomer A), and vice versa, forming a primarily hydrophobic cave surrounded by polar residues (Fig. 3).

**Figure 4:**
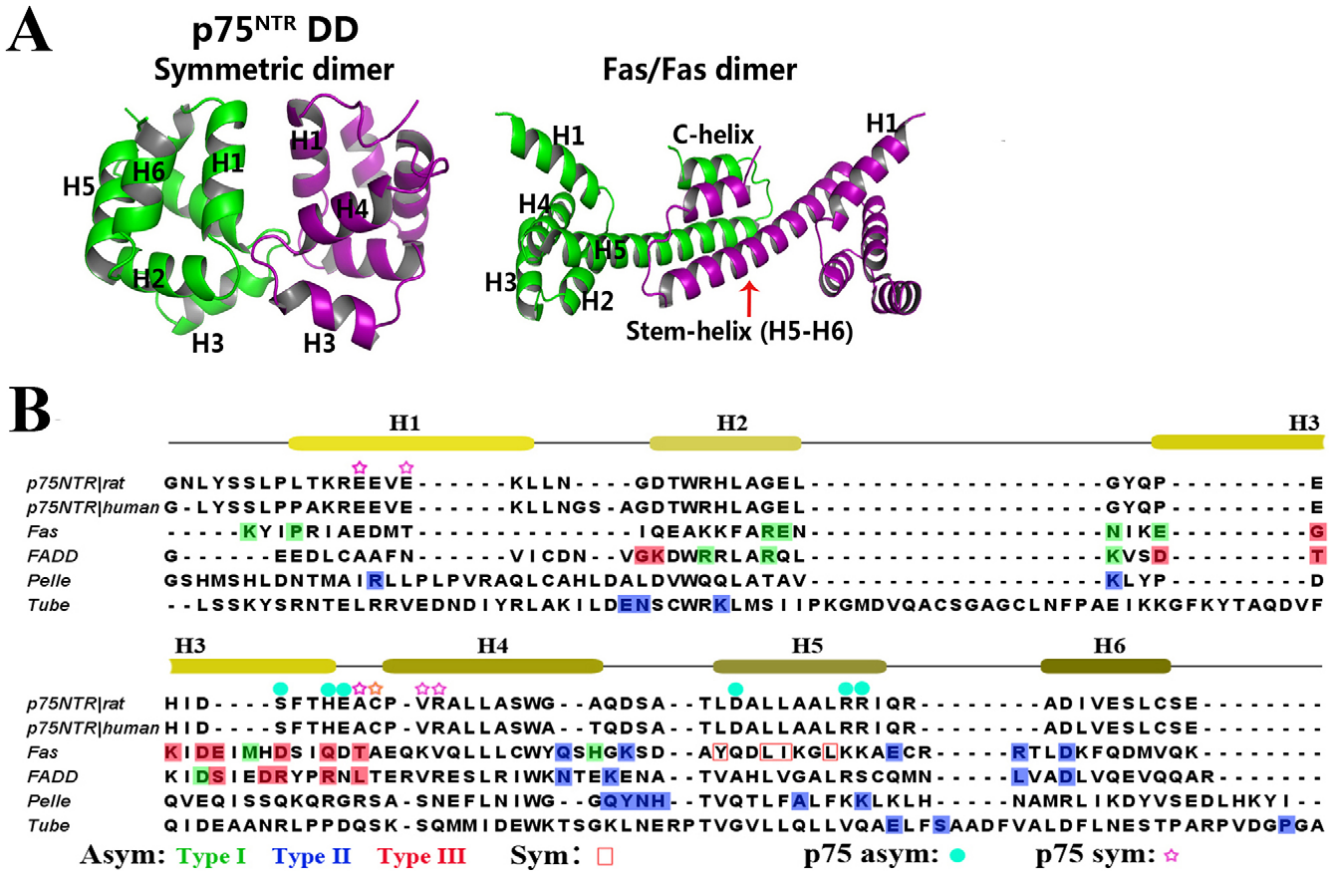
Oligomerization pattern comparison of p75^NTR^ death domain with other death domain containing proteins. (A) The symmetric p75^NTR^ death domain dimer is different from the symmetric death domain dimer observed in tetrameric Fas/FADD complex (PDB 3ZEQ). (B) Sequence alignment of p75^NTR^ death domain with other death domain containing proteins. Secondary structure of p75^NTR^ death domain is shown above the sequences, and residues involved in p75^NTR^ death domain asymmetric and symmetric dimer interfaces are indicated with cyan circle and purple pentacle above, respectively. Residues involved in three classic asymmetric interface types are highlighted as: Type I, green; Type II, blue; Type III, red; respectively. Residues important for dimerization of Fas-Fas in Fas/FADD tetrameric complex (PDB 3ZEQ) are boxed in red outline. The utmost important Cys379 for disulfide-linked dimerization of p75^NTR^ death domain was highlighted with orange pentacle.

Symmetric association of death domains also occurs in the tetrameric structure of the Fas/FADD complex [28]. However, the p75^NTR^ death domain and the Fas death domain dimerization are significantly different (Fig. 4A). First, the symmetric p75^NTR^ death domain dimer is crystallised under neutral conditions with the overall structure resembling the NMR model, while the Fas/FADD tetramer crystals are gained at pH 4.0, in which the Fas death domain is in an “open” state where the sixth helix shifts and fuses with the fifth helix to form a single long helix (stem helices), showing a large conformational change relative to the typical globular structure of its native state. Second, the p75^NTR^ symmetric death domain dimer is linked by an inter-chain disulfide bond, whereas the Fas death domain dimer is associated by non-covalent interactions between the stem helices (residues 287–318) and C-helices (a new formed helix, residues 327–334). It is noteworthy that a later NMR study suggests a different 5∶4 or 5∶5 Fas/FADD complex in solution under near physiological condition [36]. Third, the p75^NTR^ symmetric death domain dimer is likely in a self-inhibitory state, according to the Snail-Tong model [22], while the Fas death domain is in an apparently activated state when complexed with FADD. Sequence alignment also shows limited similarity of residues involving in dimeric interface between p75^NTR^ symmetric dimer and Fas-Fas dimer (Fig. 4B). These differences suggest that the symmetric p75^NTR^ death domain dimer represents a new assembly pattern for the death domain containing proteins.

### The Cys379 residue is important for dimerization of the intracellular domain of p75^NTR^

Non-reducing SDS-PAGE and mutagenesis experiments confirm the existence of an inter-chain disulfide bond in the p75DD-Fusion construct. DTT and β-ME reduce the p75DD-Fusion, and the substitution of Cys379 with serine (C379S) completely abolishes the dimerization of p75DD-Fusion (Fig. S3). Interestingly, the R382A mutation reduces the dimer fraction of p75DD-Fusion, indicating its importance in p75^NTR^ death domain dimerization (Fig. S3C). We further examine the disulfide-bond formation of p75ICD and p75CTD in solution. We find that increasing β-ME concentrations decreases the amounts of dimers of both the p75ICD and p75CTD proteins (Fig. S4). Furthermore, the C379S mutation of p75CTD largely prevents dimerization (Fig. S4C), suggesting that most of the p75CTD and p75ICD dimers in solution are disulfide-bond linked dimers.

**Figure 5:**
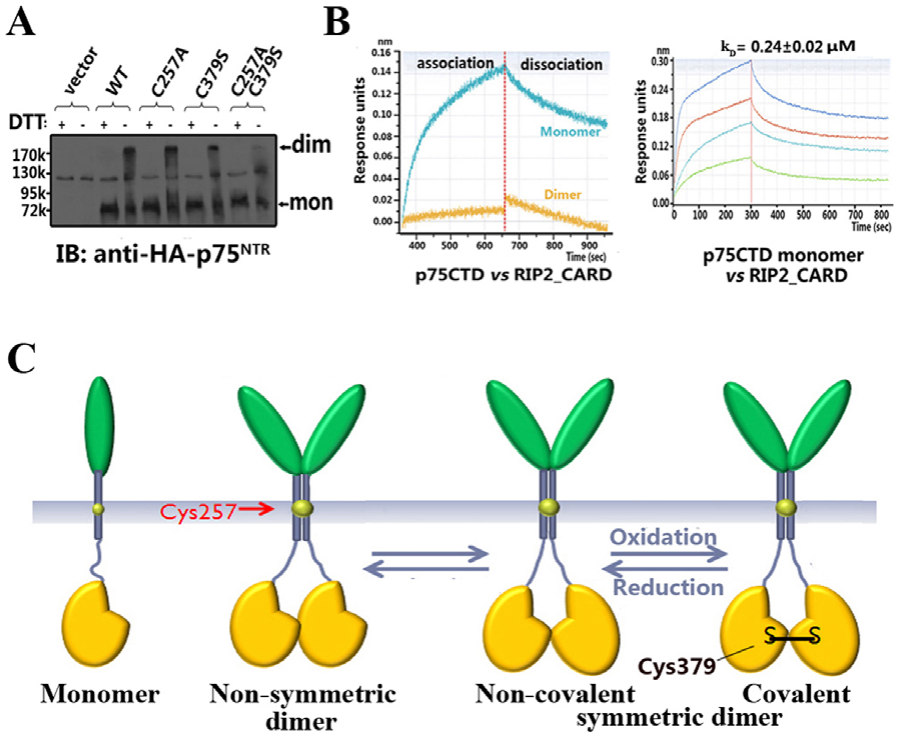
A model of p75^NTR^ signalling regulation. (A) Cell surface expression of disulfide-linked p75^NTR^ dimers (dim) and monomers (mon) in transfected HEK 293T cells by probing of p75^NTR^ immunoprecipitates treated with or without DTT. An unspecific band of MW≈130 k Da was observed in all samples and controls. (B) The binding of p75CTD monomer and symmetric dimer with the RIP2 CARD domain were analyzed by bio-layer interferometry (BLI). Binding kinetics of p75CTD monomer with the RIP2 CARD domain of different concentrations were further recorded. (C) Proposed model of p75_NTR_ activation. Different oligomerization patterns of p75^NTR^ such as monomers, non-symmetric dimers and symmetric dimers may exist simultaneously on the cell surface (based on the relative position of the intracellular domains). Regulation may result from turnover between non-symmetric dimer and symmetric dimer. An oxidation-reduction equilibrium may exist between the covalent symmetric dimer (Cys379-Cys379 disulfide bond) and the non-covalent symmetric dimer, which might confer another level of regulation through oxidative stress.

Furthermore, HEK 293T cells are transfected to test the effects of Cys379 mutation on full-length p75^NTR^ without ligand treatment. The single mutation of Cys257 or Cys379 only slightly reduces the dimer fraction, while double mutation of Cys257 (C257A) and Cys379 (C379S) nearly abolishes p75^NTR^ dimerization (Fig. 5A). This suggests that the Cys379-Cys379 disulfide bond exist in non-ligand bound cell surface p75^NTR^ dimers.

Asymmetric and symmetric p75^NTR^ death domain dimers have largely distinct surface electrostatic distributions (Fig. S5), implying an altered preference for downstream adaptors. We initially planned to determine the binding characteristics of these dimers with two downstream adaptors, receptor-interacting protein-2 (RIP2) and NRIF. However, as it was not possible to prepare a homogeneous asymmetric dimer, we measured the binding kinetics of the monomer and symmetric p75CTD dimers instead. The p75CTD monomer is obtained by double mutations of the Cys379 and Cys416 residues to serine, while the symmetric dimer is purified iteratively from the dimer fraction of the C416S mutant of p75CTD protein. RIP2 is an adaptor protein containing an N-terminal Ser/Thr kinase domain and a C-terminal caspase recruitment domain (CARD). It has been shown that RIP2 binds to p75^NTR^ via its CARD domain, though only after p75^NTR^ is activated by NGF treatment [37]. Intriguingly, the bio-layer interferometry (BLI) assay shows the RIP2 CARD domain selectively binds to the p75CTD monomer, but not the dimer (Fig. 5B), while the above-mentioned p75^NTR^-interacting peptide of NRIF could associate with both the monomer and the symmetric p75CTD dimer with relatively low affinities measured by surface plasmon resonance (SPR) (*k*_D_≈10^−5^ M; Figs. S4D and S4E), which is approximately 100-fold weaker than the binding of RIP2 CARD domain with the p75CTD monomer. These data indicate that the various oligomeric conformations of the p75^NTR^ intracellular region have different binding features.

## Discussion

As a member of the TNFR superfamily, p75^NTR^ signalling, like other non-catalytic receptors, was assumed to be mediated via ligand-induced oligomerization of intracellular domains, while the discovery of pre-existing dimers of p75^NTR^ on the cell surface provided new clues to understand the regulation of p75^NTR^ signalling [22]. Still, questions arise about whether there is equilibrium between monomers and dimers of p75^NTR^, how the dimer is assembled, and how the intracellular domains of the dimer interplay to trigger the signal cascade of p75^NTR^.

Here we described two oligomeric arrangements of the p75^NTR^ death domain, namely an asymmetric non-covalent dimer and a covalent symmetric dimer. Further analysis reveals the presence of both non-covalent dimer and Cys379-Cys379 disulfide bond linked full-length p75^NTR^ dimer on the cell surface of HEK 293T cells. It is still unknown whether the asymmetric dimer observed in the crystal structure is one form of the functionally relevant dimers on the cell surface, however, it provides some avenues for further investigation (Fig. 4B). In addition to the monomeric p75^NTR^, various oligomeric forms of p75^NTR^ such as the symmetric dimer with or without an inter-chain disulfide bond (i.e., Cys379-Cys379), and other potential non-covalent dimers may simultaneously exist on the cell surface prior to ligand binding (Fig. 5C). These multiple assembly patterns may fine-tune the regulation of p75^NTR^ activation. Though p75^NTR^ lacks intrinsic kinase activity, its activation mechanism may be similar to that of epidermal growth factor receptor (EGFR) kinase [38] in that their asymmetric and symmetric dimers correspond to different functional states.

Another level of p75^NTR^ regulation may be conferred by oxidative stress. Cysteine residues are believed to mediate cellular responses to redox conditions via both detecting oxidative changes and transducing conformational alterations in protein structure and function. A common mechanism underlying the sensitivity of cysteines to redox status is the formation of disulfide bonds. Recent studies have shown that the intracellular domain confers protection on p75^NTR^ against oxidative stress-induced apoptosis, independent of its extracellular domain or ligand binding [39], [40]. The formation of disulfide bonds between p75^NTR^ death domains suggests that the Cys379 residue may function as a redox sensor. Although the Cys379 residue of p75^NTR^ is not conserved in other death domain-containing proteins (Fig. 4B), given the dimerization or oligomerization properties of death domains, it is worthwhile to further investigate whether disulfide bonds can be formed between different death domains under certain redox conditions.

In conclusion, our results reveal the key residues involved in the formation of different p75NTR oligomers, and provide new insights into the mechanism of p75NTR activation. In addition to the significant intracellular domain rearrangement that can be induced by neurotrophin binding, the ratio of inactive covalent dimers to active non-covalent dimers could be affected by redox conditions. Binding of neurotrophins and other factors to p75NTR could then result in a shift of the equilibrium among different oligomeric states, thereby encoding multiple functional sates of p75NTR.

## Materials and Methods

### Protein expression and purification

Several constructs of the p75^NTR^ intracellular region, p75DD (residues 334–418), p75CTD (334–425) and p75ICD (280–425), were amplified respectively from a rat p75^NTR^ cDNA and constructed into pET24a vector with C-terminal His_6_ tag. Proteins were over-expressed in the *E. coli* BL21 strain and purified with Ni-NTA column and size-exclusion chromatography using a Hepes-KOH buffer (pH 7.0) containing 100 mM KCl, 5% glycerol and 1 mM EDTA. DTNB powder was dissolved in the same buffer to 2 mM concentration, and was added to protein solution at a molar ratio of 5∶1 between DTNB and proteins. After modification with DTNB, proteins were concentrated to 40 mg/mL to screen crystal growth conditions.

The p75DD-Fusion protein constructed from p75^NTR^ death domain (residues 334–418) and a 26-amino-acid sequence (residues 552–577) of the NRIF protein were fused with an 18-amino-acid linker coding for the sequence GSEFGSGSLVPRGSGSGS. These proteins were also overexpressed in the *E.coli* BL21 strain and purified in a Hepes-NaOH buffer (pH 7.5) with 100 mM NaCl, 5% glycerol and 1 mM EDTA. Proteins used for crystallization were concentrated to 20 mg/mL after DTNB treatment.

For binding tests, NRIF peptide (residues 552–577) was chemically synthesized. Human RIP2 CARD domain (residues 438–524) was constructed into pET24a vector with C-terminal His_6_ tag and overexpressed in *E. coli*. Purification of RIP2 CARD domain was similar to that of p75DD.

### Crystallization and data collection

P75DD and p75DD-Fusion protein crystals were grown at 4°C using the sitting-drop vapour diffusion method. Crystals of p75DD were obtained using 0.1 M Bis-Tris propane pH 7.0 and 1.2 M sodium citrate, while p75DD-Fusion crystals were grown in 0.1 M Bis-Tris propane pH 7.0 and 1.4 M sodium malonate pH 7.0. All crystals were harvested directly from the crystallization drop and flash-frozen in liquid nitrogen. Diffraction data for p75DD crystals were collected at 100K in-house and processed with iMosflm [41]. Data collection for p75DD-Fusion crystals was conducted at the BL17U beamline at the Shanghai Synchrotron Radiation Facility (SSRF, China) and integrated with the HKL2000 suite [42].

### Structure determination and refinement

The structures of p75DD and p75DD-Fusion were solved by molecular replacement via MOLREP [43], using the NMR structure of p75^NTR^ death domain (PDB 1NGR) as the initial search model. The residual group of the DTNB reagent (MNB) was sculpted with Sketcher [44]. The model was built with ARP/wARP and improved by cycles of manual rebuilding and refinement with REFMAC5 and Coot [45], [46], [47]. The quality of the final structure was evaluated with PROCHECK [48]. Statistics for data collection and refinement are summarized in Table 1.

**Table 1:** Data collection and refinement statistics.

	p75DD	p75DD-Fusion
**Data collection**		
Space group	*P*4_3_2_1_2	*P*3_2_
Cell dimensions		
*a*, *b*, *c* (Å)^1^	53.44, 53.44, 78.41	53.37, 53.37, 73.49
α, β, γ (°)	90.0, 90.0, 90.0	90.0, 90.0, 120
Resolution (Å)	50-2.38 (2.48-2.38)	50-2.40 (2.49-2.40)
*R*_merge_ (%)	7.1 (30.5)	5.3 (12.2)
*I/σI*	24.1 (8.1)	38.2 (14.6)
Completeness (%)	100 (100)	99.8 (100)
Redundancy	9.8 (10.1)	6.8 (6.7)
**Refinement**		
Resolution (Å)	19.6-2.38	19.6-2.40
No. reflections	4678	8699
*R*_work_/*R*_free_ (%)	21.8/25.9	22.8/28.0
No. atoms		
Protein	653	1298
Water	21	53
MNB group^2^	26	26
Average *B*-factors (Å^2^)		
Protein	23.6	37.4
Water	27.8	32.3
R.m.s deviations		
Bond lengths (Å)	0.009	0.010
Bond angles (°)	1.047	1.111
PDB ID	4F42	4F44

1 Values in parentheses refer to the outermost resolution shell.

2 MNB: one half of DTNB molecule reduced by free –SH group like cysteine residue.

### *In vivo* detection of p75^NTR^ dimerization

A full-length rat p75^NTR^ construct in a pCDNA3 vector with an N-terminal hemagglutinin (HA) epitope tag was a generous gift from Prof. Bruce Carter and Carlos Ibanez. Mutations were introduced by site-directed mutagenesis and verified by DNA-sequencing. 293T human embryonic kidney fibroblasts were cultured in DMEM. Wildtype and mutants plasmids were transiently transfected into 293T cells. After 48 h, cells were harvested and lysed in 40 mM Tris (pH 7.6) buffer containing 150 mM NaCl, 0.5% NP-40, 10% glycerol and a protease inhibitor cocktail. Cell lysates were subjected to immunoprecipitation in the lysis buffer using an anti-HA monoclonal antibody. The precipitated proteins were boiled with reduced (DTT+) or non-reduced (DTT−) SDS loading buffer before being detected by immunoblotting with anti-HA monoclonal antibody.

### Binding assays: bio-layer interferometry

The binding kinetics of the p75CTD monomer or dimer proteins to the CARD domain of RIP2 were measured by bio-layer interferometry on an Octet (ForteBio). A double cysteine residue mutant (C379S/C416S) was used as the p75CTD monomer, while the p75CTD dimer used in these experiments was the dimer of the C416S mutant. Streptavidin High Binding FA biosensors were loaded with biotinylated-p75CTD proteins in 10 mM PBS (pH 7.4) containing 0.1 mg/ml BSA and 0.002% (v/v) Tween. Loaded biosensors were first washed and transferred to wells containing CARD proteins at concentrations of 5 µM in kinetic buffer (50 mM Hepes-NaOH pH 7.5, 100 mM NaCl, 5% glycerol and 1 mM EDTA), and initial results showed no affinity for dimer proteins. We then transferred the biosensors with monomer proteins to wells containing the CARD protein at concentrations of 20 µM, 10 µM, 5 µM and 2 µM in kinetic buffer. Association and dissociation kinetics were recorded for 5 min and 10 min, respectively. Kinetic parameters (k_on_ and k_off_) and affinities (k_D_) were calculated from a non-linear fit of the data using the Octet instrument.

### Binding assays: surface plasmon resonance

Experiments to characterize the binding of p75CTD proteins and the NRIF 26-amino-acid peptide were conducted at 25°C using a Biacore 3000 SPR instrument (Biacore Life Sciences), equilibrated in running buffer containing 50 mM Hepes-KOH (pH 7.5), 100 mM KCl, 5% glycerol and 0.005% (v/v) Tween-20. P75CTD monomer and dimer proteins were coupled onto a CM-5 chip at equal molarities, and analytes were serially diluted in running buffer.

## Supporting Information

Figure S1 **DTNB stabilizes the p75^NTR^ death domain.** (A) Superimposition of different structural models of the p75^NTR^ death domain. The NMR model is shown in grey, one monomer of the p75DD crystal in green and one monomer of the p75DD-Fusion crystal in purple. (B) The MNB groups, produced by reduction of proteins with DTNB, covalently bind to the free cysteine residues of p75DD and p75DD-Fusion. (C) The polar moieties (−NO_2_ and −CO_2_) and the benzene ring of MNB interact with the surrounding residues of p75DD.
(TIF)

Figure S2 **New asymmetric interface.** The interface of p75^NTR^ death domain asymmetric dimer is different from three classic types of asymmetric interaction of death domain family, which are exemplified by Tube/Pelle complex (PDB 1D2Z) and Fas/FADD oligomer (PDB 3OQ9). The regions involved in classic asymmetric interface are indicated with blue dash circles. Residues important for the asymmetric interaction of these complexes exhibit limited conservation, as shown in Figure 4B.
(TIF)

Figure S3 **Biochemical features of the p75DD-Fusion protein.** (A) The eluted dimer portion of p75DD-Fusion was influenced by β-ME, not DTNB. (B) p75DD-Fusion proteins form dimer via disulfide bond, as determined by SDS-PAGE in the presence/absence DTT condition. (C) Mass analysis shows degradation of p75DD-Fusion (MW≈10.7 kD) observed in the crystal, but not in solution. Mutations of the symmetrical dimer interface residues of p75DD-Fusion profiled via size exclusion chromatography (D). Mutation of C379S completely abolished the dimer fraction, and the R382A mutation also decreased the dimer peak. Notably, the Pro380 residue of the H3–H4 loop region might be important to the structural stability of the entire p75DD-Fusion protein because the P380V mutant is prone to aggregation.
(TIF)

Figure S4 **Distinct surface properties of symmetric dimer and asymmetric dimer.** The N termini of the symmetric dimer (A) and asymmetric dimer (B) are colored in blue and point together towards the membrane (front view, membrane planar parallel to the paper). The distribution of the surface electrostatic potential for the symmetric dimer is different from that of the asymmetric dimer, viewed from near membrane side (top-view) and off membrane side (bottom-view).
(TIF)

Figure S5 **Features of the p75^NTR^ intracellular domain and C-terminal domain.** (A) Monomer/dimer transition of p75ICD at various concentrations of β-ME. (B) The p75CTD protein shows similar elution profile to p75ICD as the β-ME changes. (C) The cysteine mutants of p75CTD exhibit different oligomerization. Evidently, the Cys379 residue is important for p75CTD dimerization in solution. (D, E) Similar binding affinities of p75CTD dimer and monomer with NRIF peptide, measured by SPR. Almost equal molar concentrations of p75CTD monomer and dimer proteins were captured on a CM5 chip, and various concentrations of NRIF peptide (chemically synthesized) flowed over the chip in a BIACore 3000 machine.
(TIF)
