# Supplementary figures and images for: Structural Characterization of the Self-Association of the Death Domain of p75NTR
Source: PLoS One. 2013 Mar 5;8(3):e57839. doi: 10.1371/journal.pone.0057839 (PMC3589453; doi:10.1371/journal.pone.0057839)

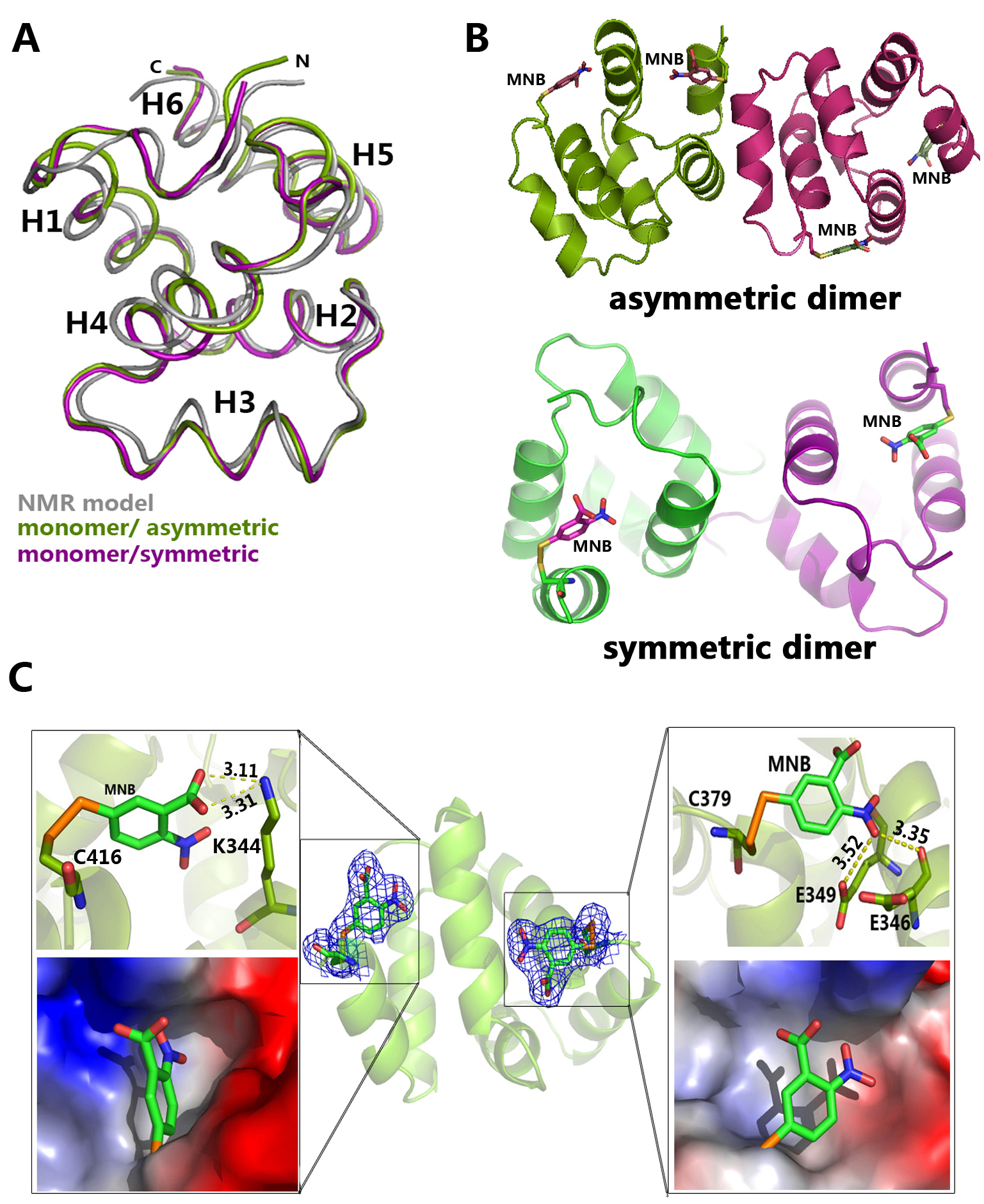

Supplement: Figure S1 — DTNB stabilizes the p75NTR death domain. (A) Superimposition of different structural models of the p75NTR death domain. The NMR model is shown in grey, one monomer of the p75DD crystal in green and one monomer of the p75DD-Fusion crystal in purple. (B) The MNB groups, produced by reduction of proteins with DTNB, covalently bind to the free cysteine residues of p75DD and p75DD-Fusion. (C) The polar moieties (−NO2 and −CO2) and the benzene ring of MNB interact with the surrounding residues of p75DD. (TIF) [file pone.0057839.s001.tif]

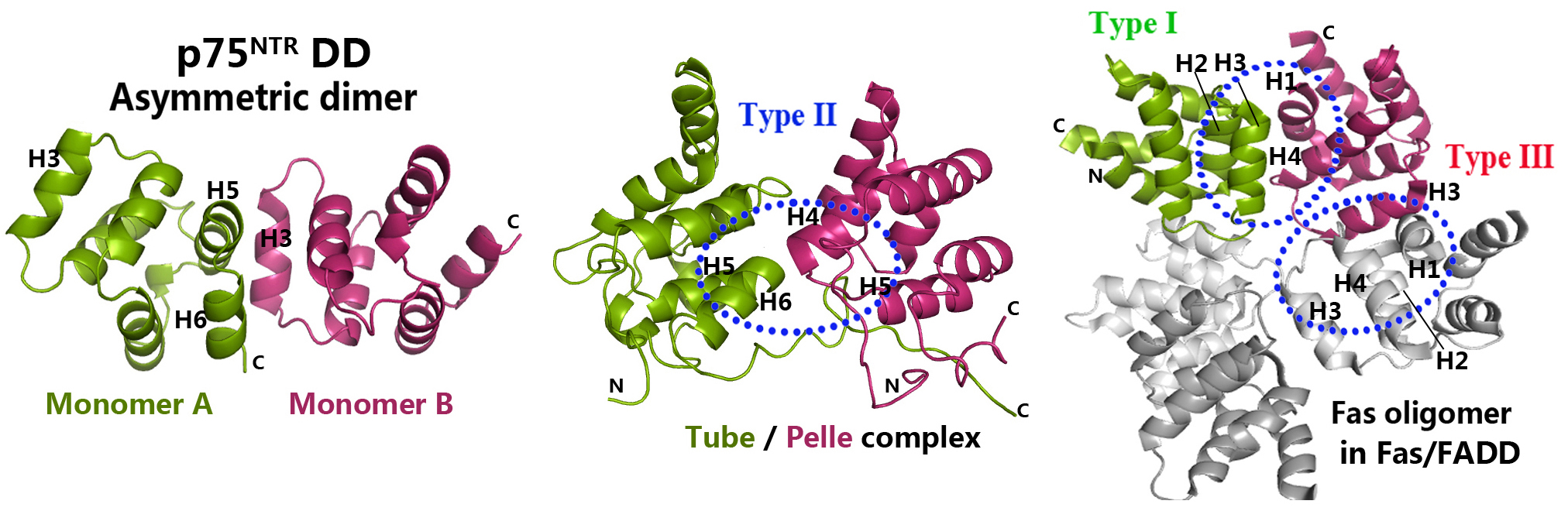

Supplement: Figure S2 — New asymmetric interface. The interface of p75NTR death domain asymmetric dimer is different from three classic types of asymmetric interaction of death domain family, which are exemplified by Tube/Pelle complex (PDB 1D2Z) and Fas/FADD oligomer (PDB 3OQ9). The regions involved in classic asymmetric interface are indicated with blue dash circles. Residues important for the asymmetric interaction of these complexes exhibit limited conservation, as shown in Figure 4B. (TIF) [file pone.0057839.s002.tif]

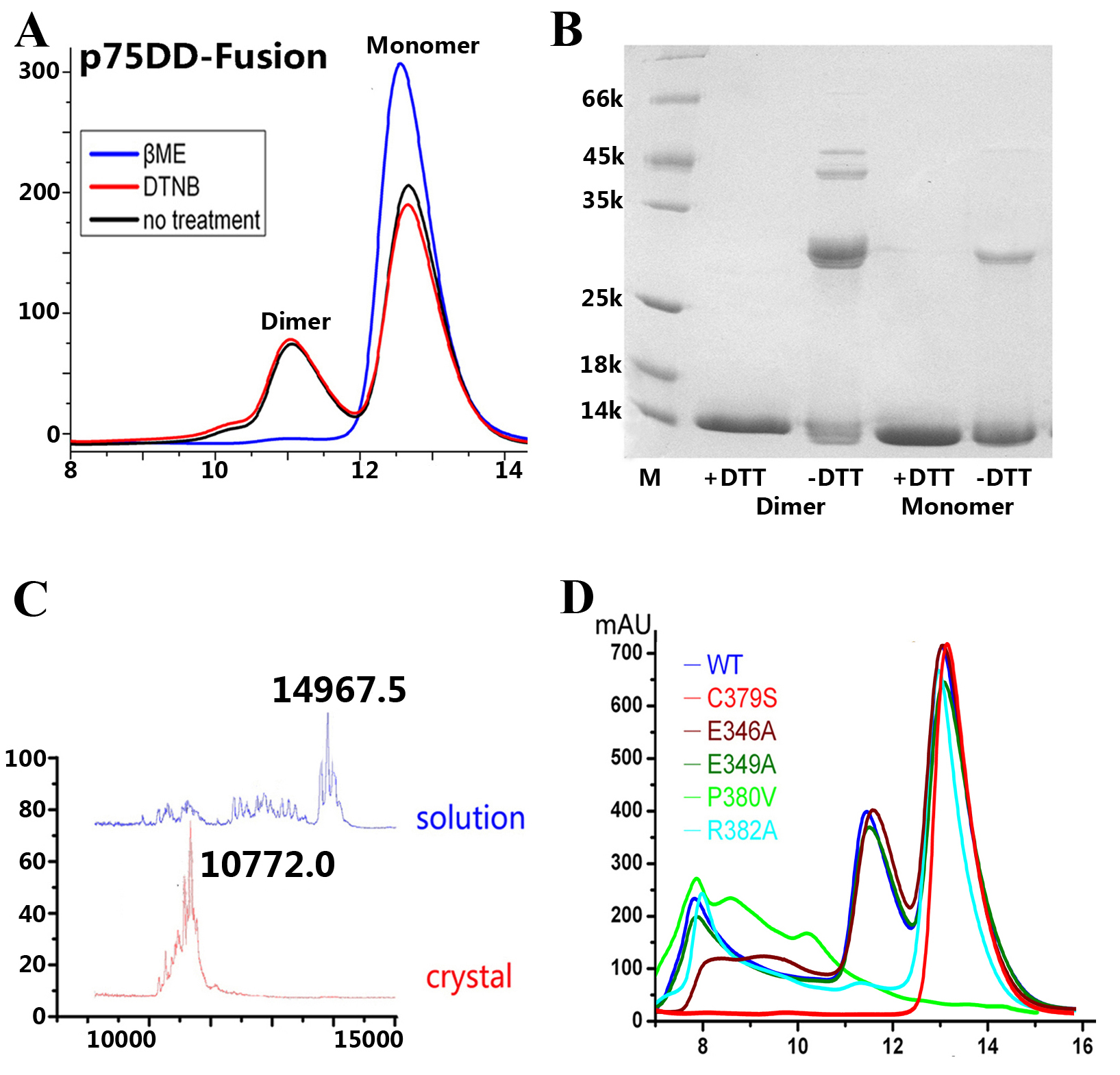

Supplement: Figure S3 — Biochemical features of the p75DD-Fusion protein. (A) The eluted dimer portion of p75DD-Fusion was influenced by β-ME, not DTNB. (B) p75DD-Fusion proteins form dimer via disulfide bond, as determined by SDS-PAGE in the presence/absence DTT condition. (C) Mass analysis shows degradation of p75DD-Fusion (MW≈10.7 kD) observed in the crystal, but not in solution. Mutations of the symmetrical dimer interface residues of p75DD-Fusion profiled via size exclusion chromatography (D). Mutation of C379S completely abolished the dimer fraction, and the R382A mutation also decreased the dimer peak. Notably, the Pro380 residue of the H3–H4 loop region might be important to the structural stability of the entire p75DD-Fusion protein because the P380V mutant is prone to aggregation. (TIF) [file pone.0057839.s003.tif]

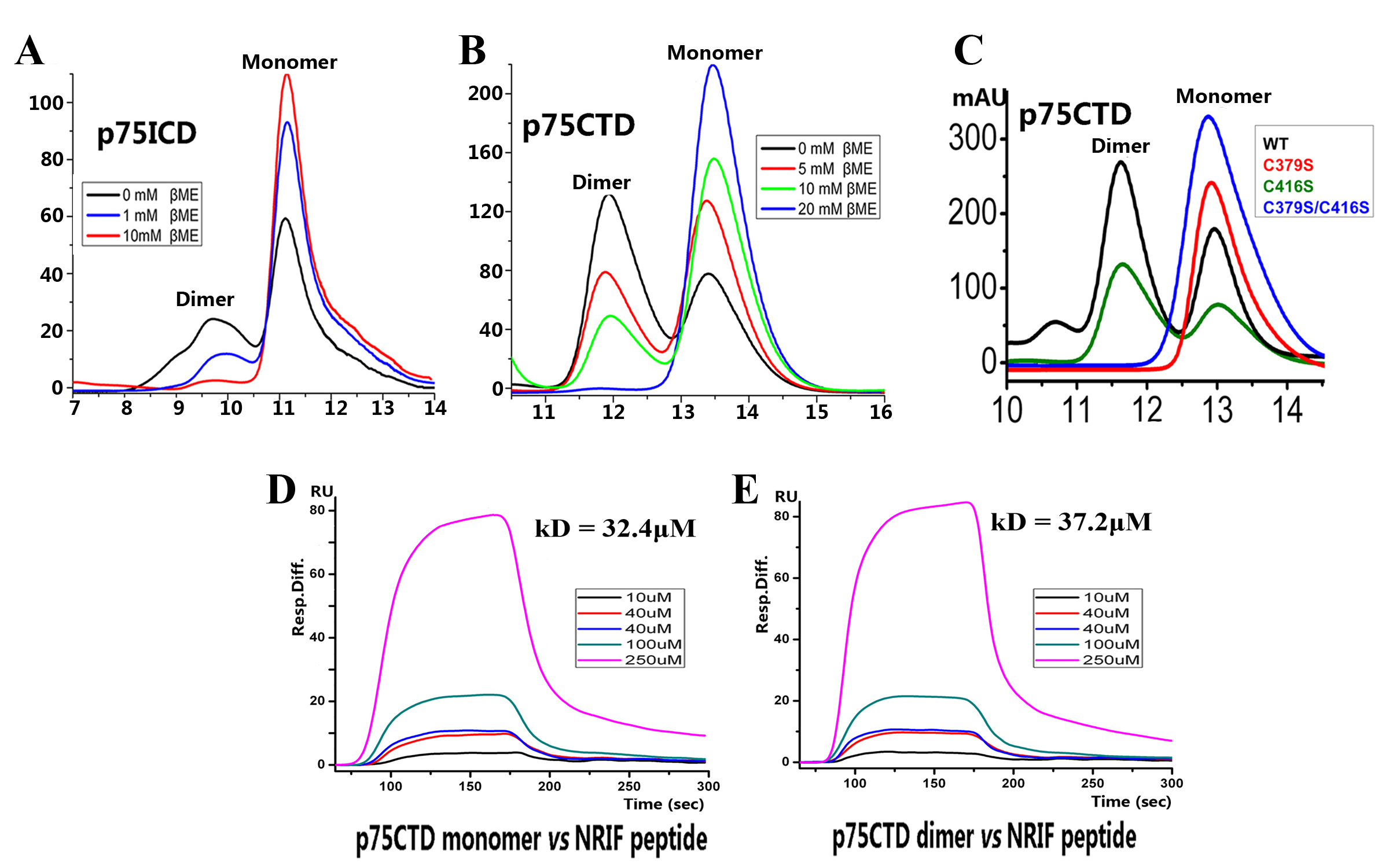

Supplement: Figure S4 — Distinct surface properties of symmetric dimer and asymmetric dimer. The N termini of the symmetric dimer (A) and asymmetric dimer (B) are colored in blue and point together towards the membrane (front view, membrane planar parallel to the paper). The distribution of the surface electrostatic potential for the symmetric dimer is different from that of the asymmetric dimer, viewed from near membrane side (top-view) and off membrane side (bottom-view). (TIF) [file pone.0057839.s004.tif]

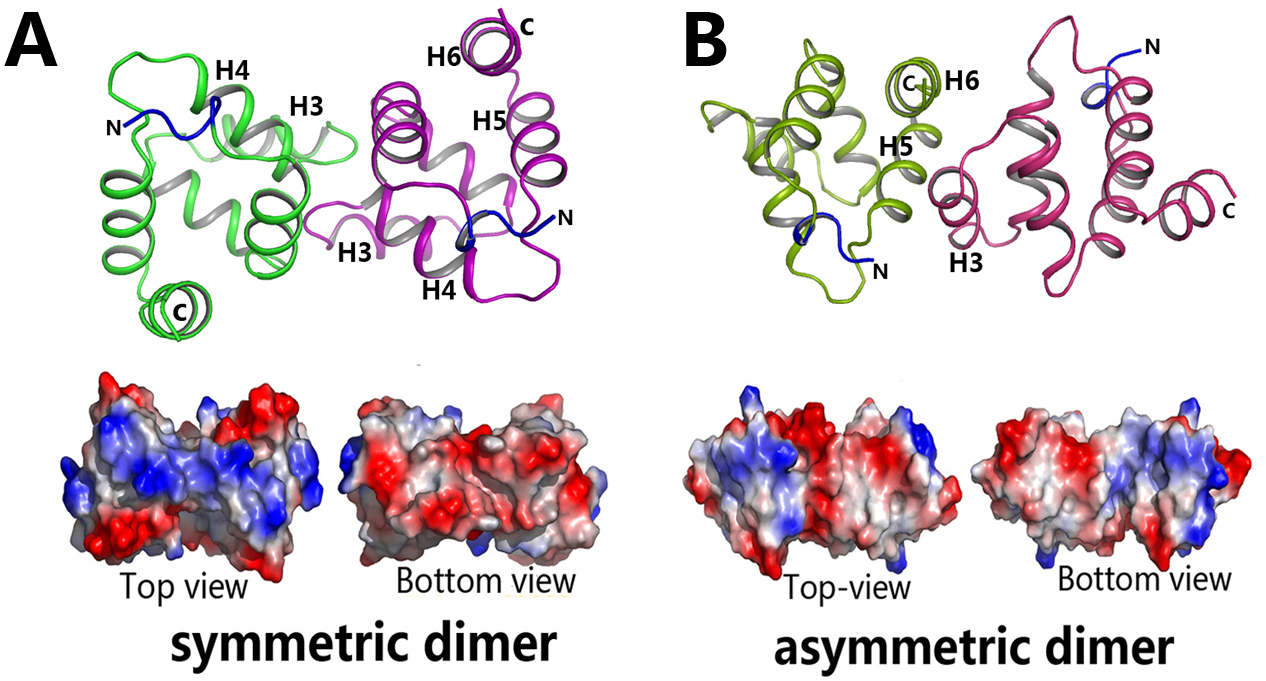

Supplement: Figure S5 — Features of the p75NTR intracellular domain and C-terminal domain. (A) Monomer/dimer transition of p75ICD at various concentrations of β-ME. (B) The p75CTD protein shows similar elution profile to p75ICD as the β-ME changes. (C) The cysteine mutants of p75CTD exhibit different oligomerization. Evidently, the Cys379 residue is important for p75CTD dimerization in solution. (D, E) Similar binding affinities of p75CTD dimer and monomer with NRIF peptide, measured by SPR. Almost equal molar concentrations of p75CTD monomer and dimer proteins were captured on a CM5 chip, and various concentrations of NRIF peptide (chemically synthesized) flowed over the chip in a BIACore 3000 machine. (TIF) [file pone.0057839.s005.tif]
